# Supplementary material for: Global analysis of suppressor mutations that rescue human genetic defects
Source: Genome Med. 2023 Oct 12;15:78. doi: 10.1186/s13073-023-01232-0 (PMC10568808; doi:10.1186/s13073-023-01232-0)
Supplement: Supplementary file 1 — Additional file 1: Fig. S1. Literature curation process. Fig. S2. Suppressor genes are important for maintaining health and cellular fitness. Fig. S3. Overlap with other interaction networks. Fig. S4. Functional connections between query and suppressor genes. Fig. S5. General mechanistic classes of suppression. Fig. S6. Query gene knockout is associated with large variation in fitness across cell lines. Fig. S7. Suppressor gene prediction. [file 13073_2023_1232_MOESM1_ESM.pdf]

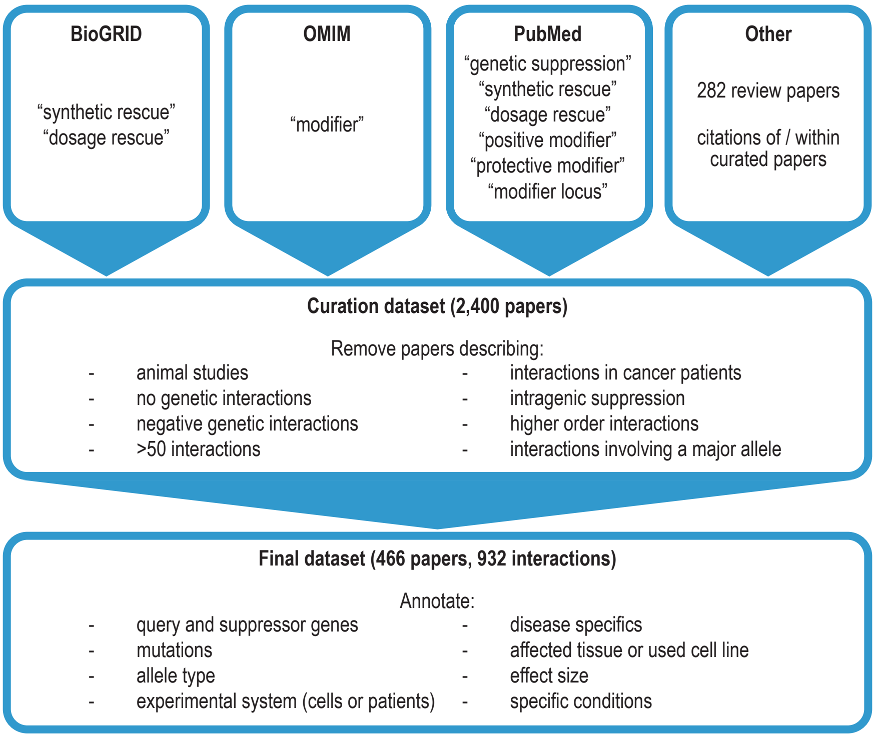

Fig. S1. Literature curation process. We examined 2,400 published papers for potential suppression interactions. Papers were derived from multiple sources: (i) the “synthetic rescue” and “dosage rescue” datasets from the BioGRID [16]; (ii) OMIM [17] data filtered for entries containing the word “modifier”; (iii) PubMed searches using the terms “genetic suppression”, “synthetic rescue”, “dosage rescue”, “positive modifier”, “protective modifier”, and “modifier locus”; and (iv) references found within the examined papers. Two genes were considered to have a suppression interaction when genetic perturbation of a query gene led to a disease, reduced survival, decreased cellular proliferation, or was otherwise associated with decreased (cellular) health, which was at least partially rescued by mutation of a suppressor gene. We excluded papers that did not describe a suppression interaction between two human genes, high-throughput studies describing >50 interactions, and interactions that were identified in cancer patients. We also excluded suppression interactions that were intragenic (occurring between two mutations within the same gene), occurred between more than two genes, or involved the major allele of either the query or the suppressor gene from the final dataset. In total, we collected 932 suppression interactions from 466 papers. From each interaction, we annotated the system in which the interaction was identified (cultured cells or patients), the query and suppressor mutations and whether these had a loss- or gain-of-function effect, the used cell line or affected tissue, the relative effect size of the suppression, whether any drugs were used, and the disease (if applicable).

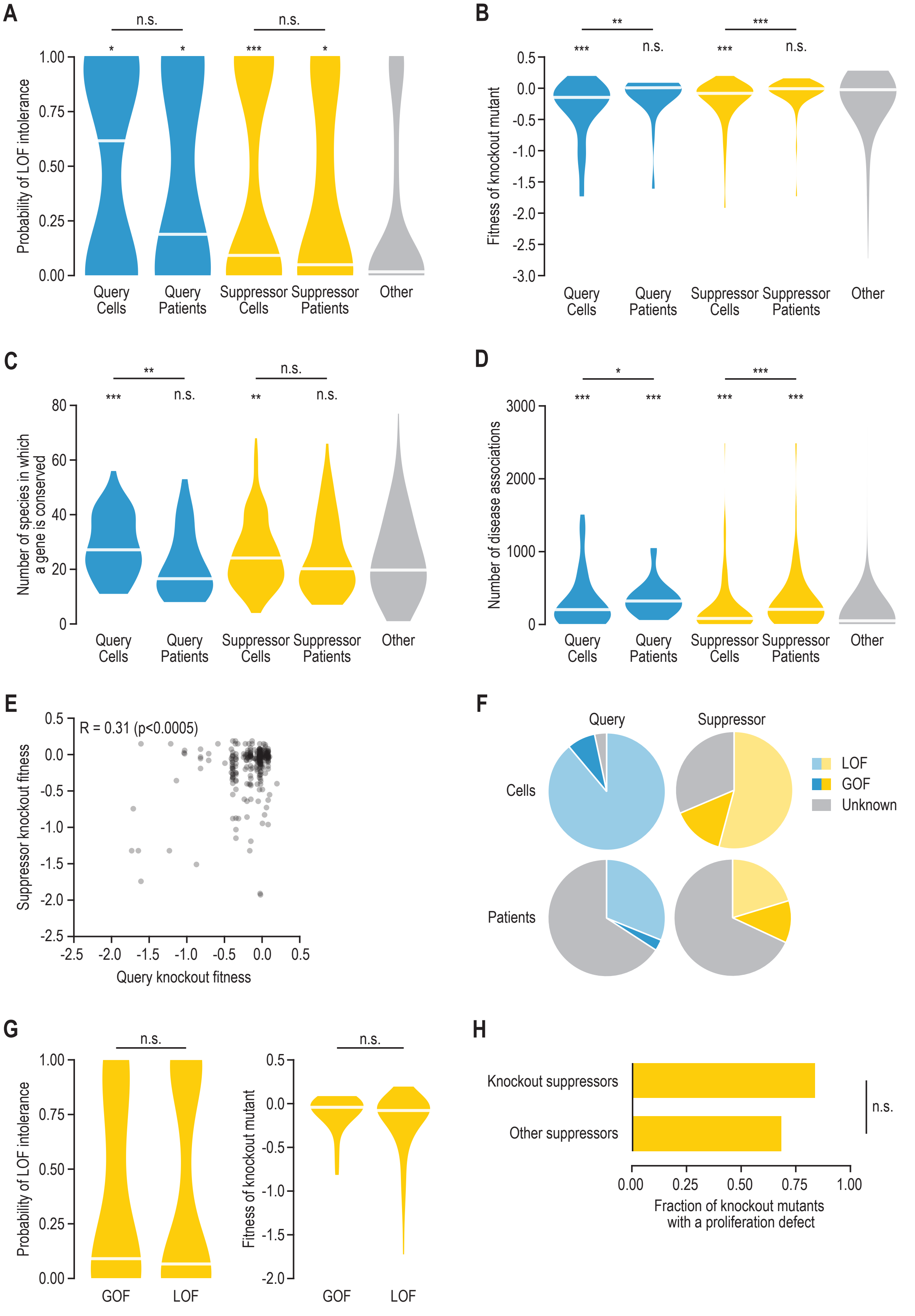

Fig. S2. Suppressor genes are important for maintaining health and cellular fitness. (**A**) Probability of loss-of-function intolerance for query genes, suppressor genes, and all other genes, based on the frequency of deleterious variants affecting the genes in the human population [25]. Query and suppressor genes were further subdivided based on whether the interactions had been described in cultured cells or patients. (**B**) Median effect of gene knockout on cell proliferation determined as the change in abundance of guide RNAs targeting a gene in pooled CRISPR-Cas9 screens across 1,070 cell lines [26, 27], for the same gene groups as in (A). (**C**) Number of species in which an ortholog of the query or suppressor gene is present. (**D**) The number of diseases that are associated with a gene in DisGeNET [29], for the same gene groups as in (A). (**E**) Median effect of gene knockout on cell proliferation as in (B) for query-suppressor gene pairs. The Pearson correlation coefficient and corresponding p-value are indicated. (**F**) The fraction of query and suppressor genes that have loss-of-function (LOF), gain-of-function (GOF), or unknown modes of action. (**G**) Probability of loss-of-function intolerance and the effect of gene knockout on cell proliferation as in (A) and (B), for suppressor genes that carry loss-of-function or gain-of-function mutations. (**H**) The fraction of knockout mutants that have a negative mean fitness across 1,070 cell lines [26, 27] for suppressor genes that were identified through gene knockout in cultured cells or for all other suppressor genes. Statistical significance compared to the "Other" group (A-D) or between indicated groups (A-D, G-H) was determined using Mann-Whitney U tests. n.s. = not significant, * p<0.05, ** p<0.005, *** p<0.0005. Horizontal lines in violin plots: median.

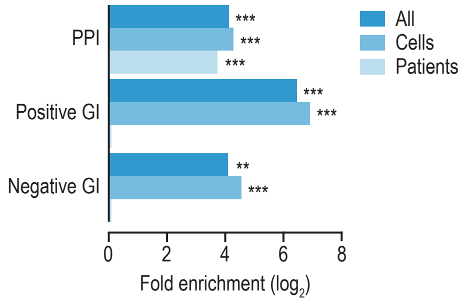

Fig. S3. Overlap with other interaction networks. Fold enrichment for overlap of suppression interactions with protein-protein interactions (PPI), or with positive and negative genetic interactions (GI), either for all suppression interactions, or for interactions identified in cultured cells or in patients only. Fisher's exact tests were performed to determine statistical significance of the results. * $p<0.05$, ** $p<0.005$, *** $p<0.0005$.

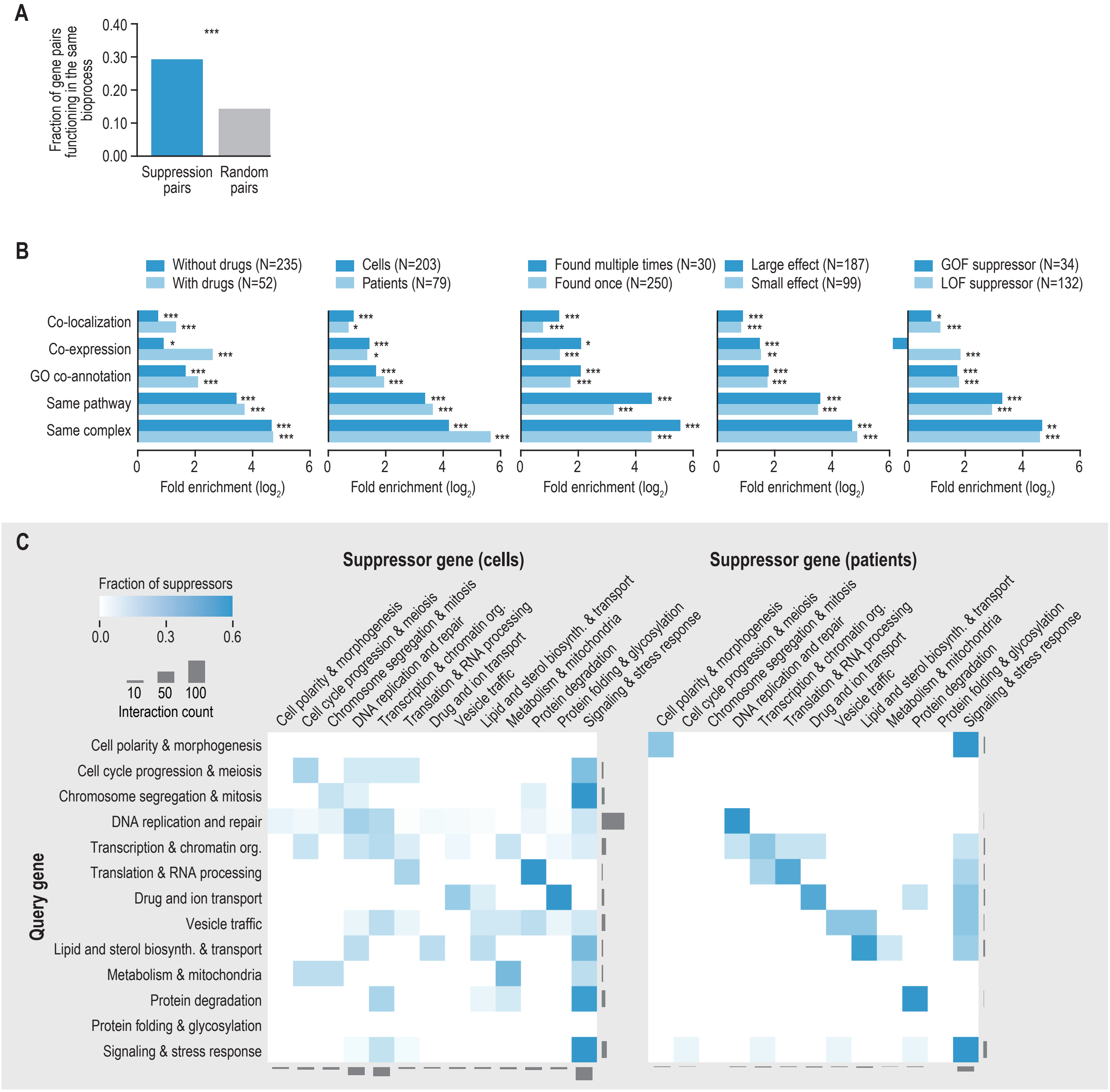

Fig. S4. Functional connections between query and suppressor genes. (**A**) Fraction of query-suppressor or random gene pairs sharing a biological process annotation. (**B**) Fold enrichment for co-localization, co-expression, GO co-annotation, same pathway membership, and same complex membership for different subsets of suppression interactions. The number of interactions in each subset is indicated in the legends. Note that the total number of interactions varies per analysis, because some interactions may have neither or both annotations (i.e. an interaction may have been identified both in the presence and in the absence of a drug). (**C**) Frequency of suppression interactions connecting genes within and across indicated biological processes for interactions identified in cultured cells (left) or in patients (right). Color reflects the fraction of suppressor genes belonging to a particular biological process for all interactions involving query genes annotated to a given biological process. The total number of suppression interactions involving genes annotated to a particular process is indicated. Fisher's exact tests were performed to determine statistical significance of the results in (A) and (B). * $p<0.05$, ** $p<0.005$, *** $p<0.0005$.

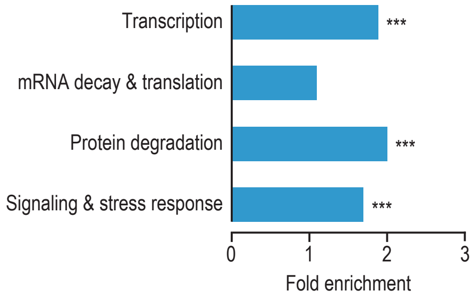

Fig. S5. General mechanistic classes of suppression. Fold enrichment of suppressor genes for GO terms associated with the indicated general mechanisms of suppression. Fisher's exact tests were performed to determine statistical significance of the results. * $p<0.05$, ** $p<0.005$, *** $p<0.0005$.

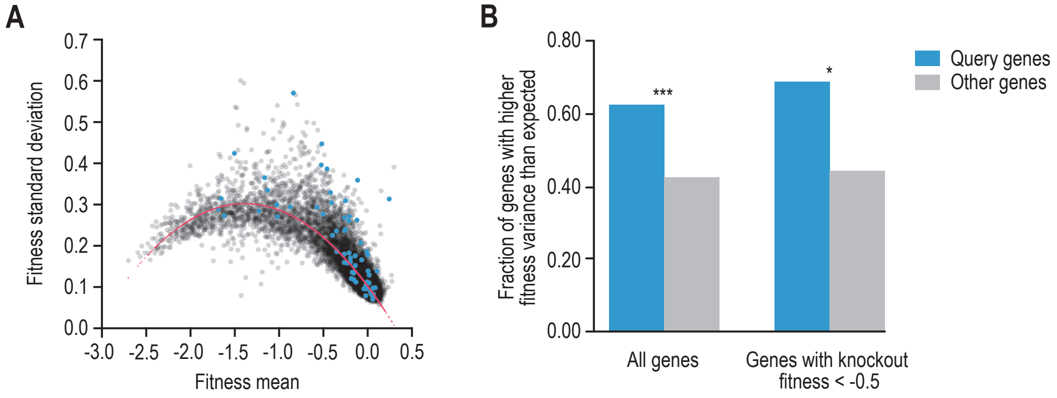

Fig. S6. Query gene knockout is associated with large variation in fitness across cell lines. (**A**) Plotted are the mean fitness of a knockout mutant across 1,070 cell lines against the fitness standard deviation [25, 26]. Each data point represents a gene, query genes are highlighted in cyan. Pink = the model that was fit to the data. (**B**) Fraction of genes that have a higher variance in fitness across cell lines than expected by chance given the model shown in (A), for either all genes or for genes with an average knockout fitness across cell lines <-0.50. Statistical significance was determined using one-sided Fisher's exact tests. * $p<0.05$, ** $p<0.005$, *** $p<0.0005$.

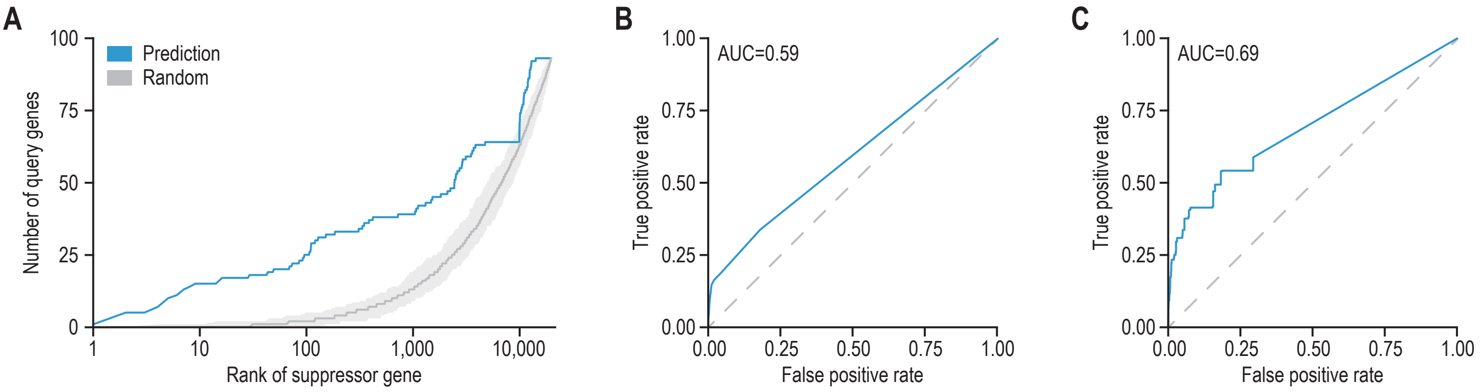

Fig. S7. Suppressor gene prediction. (**A**) A suppressor gene prediction model we previously developed in yeast based on the strong functional connection between query and suppressor genes [5], was used to predict suppressors for human query genes. For each query gene, the rank of the validated suppressor gene(s) was determined on both a random gene list and on a list of genes ranked by the likeliness of being a suppressor gene using the prediction algorithm. The rank of the validated suppressor gene was plotted against the number of query genes that interacted with a suppressor gene with that rank. (**B**-**C**) Evaluation of suppressor prediction model performance. The true-positive rate was plotted against the false-positive rate for the functional prioritization model (B) and the random forest model (C).
